# A computation analysis with heat and mass transfer for micropolar nanofluid in ciliated microchannel: With application in the ductus efferentes

**DOI:** 10.1016/j.heliyon.2024.e39018

**Published:** 2024-10-05

**Authors:** Ali Imran, Hanadi Alzubadi, Mohamed R. Ali

**Affiliations:** a Department of Mathematics, COMSATS University Islamabad, Attock Campus, Kamra Road Attock Punjab, Pakistan; b Department of Mathematics, Umm AL-Qura University, Makkah, Saudi Arabia; c Faculty of Engineering and Technology, Future University in Egypt New Cairo 11835, Egypt

**Keywords:** Ciliary flow, Ductus efferentes, Micropolar fluid, Bvp4c technique

## Abstract

Inherited heat enhancement capabilities and their significance in the field of medical sciences and industry make nanofluids the focus of research nowadays. Furthermore, due to the remarkable advancements in bionanotechnology and its significance in biomedical fields such as drug delivery systems, cancer tumor therapy, bioimaging, and many others, it has emerged as a key research area. Contribution of cilia for the flow in ductus efferentes of human male reproductive tract is elaborated. A novel mathematical scheme is presented for the heat and mass transfer of MHD micropolar nanofluid transport in an asymmetric channel lined with cilia. The pertinent equations of nanofluid transport are exposed to lubrication approximation theory and solution for the physical problem is examined with efficient bvp4c technique in MATLAB. Fluid rheology is explored with the variations of different transport parameters like Hartmann number, Grashof number, Brownian motion, buoyancy, thermophoresis and Darcy number. It is reported that nanofluid transport is affected with rise in the Lorentz force and show reverse behavior with rising permeability. The temperature of the nanofluid in ciliated microchannel is raised with enhanced value of Hartmann number, Grashof number, Prandtl number, and Darcy number while diffusion phenomenon of nanofluid is slowed down with these parameters. Spinning motion of the nanofluid is enhanced with Grashof number and slow down with nanoparticle Grashof number and different behavior is recorded for Darcy and viscosity parameters in different flow regime. Reported investigation presents crucial findings for ciliary transport of micropolar nanofluid and tackled with appropriate selection of micropolar parameter, Brownian motion parameter, thermophoresis and Grashof number. Moreover, this investigation will be handful for cilia-based actuators which work as micro-mixers in controlling the flow in minute bio-sensors and may prove their worth in micro-pumps employed in various drug-delivery systems.

**Table:** Nomenclature

**Alphabetic Letter**			
$\left(\overset{˘}{X},\overset{˘}{Y}\right)$	Axial and transverse directions(m)	$m_{1}+m_{2}$	Width of the channel (m)
$\left({\overset{˘}{U}}_{0},{\overset{˘}{V}}_{0}\right)\text{,}$	Axial and transverse velocity components ($ms^{-1}$)	c	Wave speed ($ms^{-1}$)
${\overset{˘}{T}}_{0}$	Temperature at lower wall (K)	$D_{a}$	Darcy number
${\overset{˘}{C}}_{0}$	Concentration at lower wall ($kg/m^{3}$)	$l_{1},l_{2}$	Magnitudes of the wave (m)
$W_{0}$	Microrotation at lower wall (rad)	Τ	Stress tensor ((N/m²)
$\left({\overset{˘}{U}}_{1},{\overset{˘}{V}}_{1}\right)\text{,}$	Axial and transverse velocity components at upper wall ($ms^{-1}$)	k	Viscosity constant
${\overset{˘}{T}}_{1}$	Temperature at upper wall (K)	$k_{1}$	permeability (W/m/K)
${\overset{˘}{C}}_{1}$	Concentration at upper wall ($kg/m^{3}$)	$c_{p}$	Specific heat (J/(kg·K)).
$W_{1}$	Microrotation at upper wall (rad)	$D_{B}$	is the Brownian motion parameter
T	Temperature of the fluid (K)	$B_{0}$	Constant magnetic field($Wb/m^{2}$)
$D_{T}$	is the thermophoretic diffusion coefficient	C	Concentration of nanoparticles ($K/m^{3}$)
u	Dimensionless axial velocity	M	Magnetic number
Pr	Prandtl number	$G_{m}$	Nanoparticle Grashof number
Re	Reynolds Number	Br	Brinkmann number
Gr	Grashof Number	Nt	Thermophoresis parameter
$N_{b}$	Brownian motion parameter		
**Greek Letter**			
λ	Wavelength (m)	$\lambda_{1},\lambda_{2}$	ratio of relaxation to retardation times, retardation time
α	is the thermal diffusivity	$\rho_{f}$	Density of the fluid ($kg/m^{3}$)
σ	is the electrical conductivity (S/m).	$\rho_{p}$	Density at constant pressure ($kg/m^{3}$)
$\zeta_{t}$	thermal expansion coefficient	Ω	Dimensionless nanoparticle concentration
$\zeta_{c}$	viscosity at constant concentration	δ	Wave number
θ	Dimensionless temperature	μ	Is the dynamic viscosity (Pa·s)

## 1 Introduction

The ductus efferentes comprise of several microvessels attaching testis to epididymis [1]. The composition of these vessels consists of epithelium layers which are reinforced by smooth muscle and adjacent tissues. These vessels performed physiological processes of great significance, for instance narrated vessels transport sperm via rete testis to epididymis and take excess quantity of biological fluid arising from rete testis. Because of the significance of the above narrated functions spermatozoa concentration raised to manifold. Ductus efferentes consists of ciliated and some other sort of cells. The surface of the cilia is surrounded with ciliated cells with the lumen of duct and thickly enclosed, microvilli is supported by non-ciliated cells.

Cilia appear to appendages which occur on the free surface of pertinent cells. Cilia have been revealed in nearly all animal kingdom since they play crucial roles in many physiological operations like alimentation, circulation, respiration and more importantly in the ductus efferentes of human male reproductive tract [2]. Rivera [3] based upon the data of various aquatic species presented his observations which may be summarizes as: (i) in a specific tissue all cilia beat quite systematically (ii) the beat of individual cilium is well managed (iii) an appreciable metachronal rhythm came to existence. A metachronal wave is a sequential sort of movement developed due to lashing of cilia. Blake [4] presented a theoretical model tackling the ciliary motion in an array of flexible long slender bodies. Blake [5] investigated the 2-D swimming motion of the protozoan by using spherical envelope and explored the swimming motion through ciliated cylinder. Mucus transport in the mammalian trachea focusing on Newtonian mechanics has been discussed [6]. Miller [7] reported mucus transport phenomenon using mechanical simulation of ciliary motion in the trachea. Studies on protozoology and mucus dynamics in the respiratory tract provide a concept of locomotion in protozoa and also gave in-depth information about the movement of some particles in the respiratory tract. It is extremely crucial to mentioning here that limited literature is available about the role of cilia and the metachronal rhythm for fluid transport in efferent ducts. Inspiration of the reported study arise due to availability of little knowledge pertaining to the fluid transport in the ductuli efferentes of male reproductive tract and the possible consequences of cilia on ovum movement and sperm transport in fallopian tubes.

Nanofluids, because of their numerous applications in industries and in medical science, have gathered the attention of many researchers [8]. Nanofluids are formed with the suspension of nanoparticles having size less than 100 nm. Nanofluids are the eyeball of researchers because of their astonishing heat transfer capabilities even at low nanoparticles concentration [9]. Various researchers explored the hydrodynamics of protozoa using cilia lash for locomotion. Exploration of in-depth knowledge of the nanofluid behavior is subject of many research articles nowadays due to their huge significance in general industries, nuclear reactors, transport industry, electronics, biological sciences, and in food industry where efficient and economical heat transfer is required.

The astonishing characteristic of nanofluids includes, it may be used for heat enhancement over large area between particles and fluids, exceptional diffusion ability with a prevalence of Brownian motion of the particles, have low pumping power requirements in comparison to traditional liquids. Newtonian fluid characteristics may be recovered from micropolar fluids, which are simply known as micropolar fluids. Micropolar fluids possesses microstructure having asymmetrical stresses. Akbar et al. [9] presented a study for nanofluid peristaltically induced flow by focusing the temperature and nanoparticle concentrations by employing the HPM. The effects of Joule heating for MHD flow of nanofluid in a channel has been reported [10] for the case complaint boundaries. Asgar et a [11]. introduced the impact for a peristaltic transport of blood hybrid Cu−Au nanofluid through a ciliated micro-vessel with the buoyancy and Lorentz force variations. Significance of blood transport coupled with titania Cu−Au nanoparticles through the varying cross sectional micro-vessel carpeted with cilia have been reported [12] and explored their diverse applications in the biomedical and physiological domain. Karmaker et al. [13] analyzed electric double flow of blood-oriented transport for tetra-hybrid nanoparticles in an endoscopic arterial geometry. Paul et al. [14] reported the electro-pumping dynamics of Phan–Thien–Tanner blood flow nanofluid in the ciliated artery. Electrically managed soft-robotic ciliated epidermis having various polypyrrole driven bending actuators have been reported [15]. Electro-osmotic flow of rheological fluid within a channel carpeted with cilia have been reported [16,17] with activation energy axioms. Cilia induced multiphase transport of non-Newtonian rheological fluid for 2-D and 3-D channel by implementing EMHD have been investigated [18,19]. Khan et al. [20] evolved a mathematical model for rheological fluid transport in a symmetric and obtained an analytical solution using perturbation technique. Shakib et al. [21] exhibited a theoretical model for mixed convective of Casson fluid in a ciliated curved channel. Abbas et al. [22] inspected the mixed convection of ternary hybrid nanofluid in a ciliated curved channel. Various key flow features with mass and heat transfer of multilayered flow of rheological fluid due to metachronal ciliated channel have been reported [23,24] along with pertinent applications in the field of biomedical sciences, bioengineering, and medical devices engineering. Fatima et al. [25] investigated the heat and mass transport for a two-layered flow of Newtonian fluid developed by cilia lash by considering buoyancy forces.

The growing significance of nanofluids has attracted many researchers [26,27]. Nanofluids are engineered by mingling nanoparticles, nanofiber, nanosheets, nanotubes, or droplets into a base fluid [28]. These fluids exhibit excellent thermos-physical axioms like heat conduction/diffusion, and good thermal exchange behavior as compared to other conventional base fluids for instance water or oil [29]. These days there are enormous applications of heat and mass transfer in biological engineering, and in different industrial settings using cilia transport, also heat transition has role in blood flow, heat creation, biomass transfer and in the treatment hypothermia problem. Goher et al. [30] elaborated the heat transfer analysis for two distinct boundary layer flows of Newtonian fluid. The impact of nonlinear thermal radiation coupled with slip, and Joule heat for MHD pulsating transport along with entropy generation has been reported [31]. The physical aspects of mass and heat transfer for Casson nanofluid and hybrid nanofluid in a two-layer transverse channel has been discussed [32]. Turabi et a [33]. investigated the fluid rheology for convective nanofluid and ternary hybrid nanofluid in a microchannel. Amin et al. [34] explored the boundary layer transport of hybrid nanofluid through horizontal plates of infinite length using MHD, magnetic and viscous dissipation. The non-Newtonian aspect of blood flow has been interrogated [35] by focusing incompressible, laminar, flow with viscous dissipation phenomenon.

Nanofluids play absolutely vital role in heat enhancement and have great significance in the field of medical sciences and industry. Further due to rapid development in bio nanotechnology and its significance in biomedical, in drug delivery systems, cancer tumor therapy, in bioimaging, and many others, emerged as a key research area. Cilia have a significant role in many physiological processes and ciliary movement helps to transport fluids along and enable sperm cells to play their part. In this work heat and mass transfer for MHD micropolar nanofluid transport in an asymmetric channel by incorporating various physical effects is investigated.

The thermophysical properties of gold nanoparticles are absolutely crucial in the context of numerous applications, like in biomedical science, optical, and electronic domains. The thermophysical properties [39] vary with size, shape, and composition of the nanoparticles. Gold nanoparticles give impetus to thermal conductivity due to size properties and the manifestation of surface atoms. The gold nanoparticles when added to base fluid substantially enhance thermal conductivity. Which relies on the concentration, size, shape, and distribution of the nanoparticles. The specific heat capacity of gold nanofluids is usually less than that of the base fluid. The gold nanoparticles usually enhance the viscosity of the base fluid, which depends on the nanoparticle concentration, size, shape, level of interactions between particles. Thermal diffusivity, which is defined as heat conduction within the material, is boosted with gold nanofluids, due to the enriched thermal conductivity because of the gold nanoparticles. Because of the enhanced thermal conductivity and thermal diffusivity [40], gold nanofluids are frequently used to boost heat transfer in cooling systems, heat exchangers, and other thermal controlling applications. The flow behavior of gold nanofluids may vary with the base fluid. For example, with high nanoparticle concentrations, the fluid may exhibit more viscous or may behave like non-Newtonian.

The micropolar fluid model embarks on a powerful framework for analyzing and investigating effects of biological fluids. By incorporating nanofluid, this model exhibits more accurate descriptions and projections, which are extremely beneficial in numerous applications in biomechanics, range from blood flow investigations to engineering manipulations of medical devices and treatment approaches. The usage of micropolar fluid dynamics in biomechanics is a vibrant area of research domain, contributing to theoretical and practical aspects of biomedical engineering. Inspired by huge applications of biological fluid, striving is made to embark novel heat and mass transfer for MHD micropolar nanofluid transport in an asymmetric channel carpeted with cilia with by Brownian motion, buoyancy, and thermophoresis effects. A crucial mathematical model evolved for the physical problem and the pertinent equations of motion are simplified by focusing lubrication approximation theory. The solution for momentum, temperature, concentration, and microrotation equations of motions for micropolar nanofluid is explored in MATLAB using renowned bvp4c technique.

Novel flow features are reported to provide a more comprehensive insight of fluid transport as a function of cilia. We employ ciliary pumping characteristics for the exploration of hydrodynamics of protozoa which rely on ciliary motion. Finding of the reported investigation will be handful for cilia-reliant actuators which work as micro-mixers in controlling the flow in minuet biosensors and may prove their worth in micro-pumps employed in various drug-delivery systems.

## 2 Mathematical flow demonstration

**Fig. 1:**
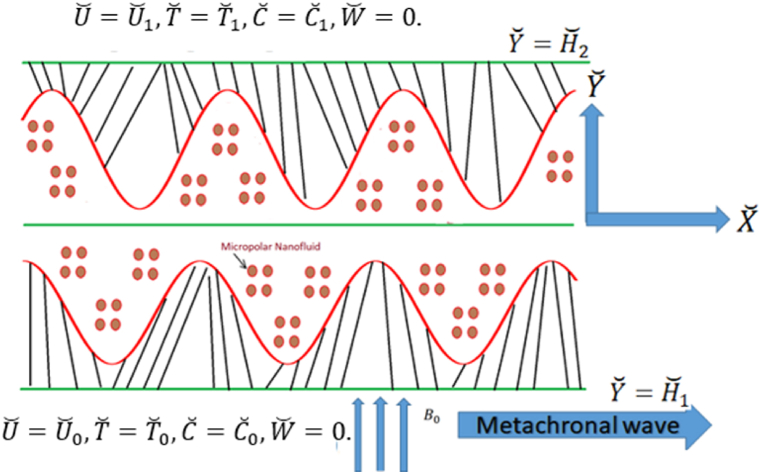
The flow scheme for ciliated microchannel.

Let us contemplate cilia driven flow of micropolar nanofluid in an asymmetric horizontal channel which comprise of gold nanoparticles. It is assumed that a metachronal propagates to right hand side of the microchannel with wave speed c. We want to elaborate the fluid transport features in the microchannel by relying on ciliary lashing and metachronal wave. The fluid inside the channel is assumed to be electrically conducting non-Newtonian micropolar fluid. The flow dynamic scheme is designed by focusing rectangular coordinate system $\left(\overset{˘}{X},\overset{˘}{Y}\right)$, where $\overset{˘}{X}$ elaborates the axial direction and $\overset{˘}{Y}$ is transverse to it. The flow scheme is exhibited for the physical problem in Fig. 1. A uniform magnetic field is anticipated in $\overset{˘}{Y}$ direction. The values of velocity, nanofluid temperature, respective concentration, and microrotation at upper wall are $\left({\overset{˘}{U}}_{0},{\overset{˘}{V}}_{0}\right),{\overset{˘}{T}}_{0},{\overset{˘}{C}}_{0},W_{0}$ and lower wall is held at $\left({\overset{˘}{U}}_{0},{\overset{˘}{V}}_{0}\right),{\overset{˘}{T}}_{1},{\overset{˘}{C}}_{1},W_{1}$.

The description of the asymmetric ciliated channel may be demonstrated mathematically as [[36], [37], [38]].

(1) $${\overset{˘}{H}}_{1}=m_{1}+l_{1}\;\cos\frac{2\pi }{\lambda }\left(\overset{˘}{X}-c\overset{˘}{t}\;\right)$$

(2) $${\overset{˘}{H}}_{2}={-m}_{2}-l_{2}\;\cos\frac{2\pi }{\lambda }\left[\left(\overset{˘}{X}-c\overset{˘}{t}\;\right)+\phi \right]$$

Where $\overset{˘}{X}$ and $\overset{˘}{Y}$ are axial and transverse the direction of the propagating metachronal wave, $m_{1}+m_{2}$ represents width of the microchannel, $\lambda ,c,l_{1},l_{2}$ reflects wavelength, speed, and amplitudes of propagating metachronal wave, ϕ signifies phase difference and varies between 0≤ϕ≤π. The above narrated constraints satisfy the relation $l_{1}^{2}+l_{2}^{2}+2l_{1}l_{2}\;\cos\;\phi \leq {\left(m_{1}+m_{2}\right)}^{2}$.

The extra Cauchy stress tensor Τ for Micropolar fluid [34] is defined underneath as:

(3) $$Τ=-\overset{˘}{P}I+\frac{\mu }{1+\lambda_{1}}\left[\dot{\gamma }+\lambda_{2}\frac{d\dot{\gamma }}{dt}\;\right]$$

The pertinent continuity, momentum, temperature, concentration, and microrotation equations [34,35] of motions for micropolar nanofluid for the velocity field $V=\left[\overset{˘}{U}\left(\overset{˘}{X},\overset{˘}{Y},\overset{˘}{t}\right),\overset{˘}{V}\left(\overset{˘}{X},\overset{˘}{Y},\overset{˘}{t}\right),0\;\right]$ are

(4) $$\frac{\partial \overset{˘}{U}}{\partial \overset{˘}{X}}+\frac{\partial \overset{˘}{V}}{\partial \overset{˘}{Y}}=0\text{,}$$

$$\rho_{f}\left[\frac{\partial }{\partial \overset{˘}{t}}+\overset{˘}{U}\frac{\partial }{\partial \overset{˘}{X}}+\overset{˘}{V}\frac{\partial }{\partial \overset{˘}{Y}}\right]\overset{˘}{U}=\frac{\partial }{\partial \overset{˘}{X}}\left[-\overset{˘}{P}+{\overset{˘}{S}}_{\overset{˘}{X}\overset{˘}{X}}\right]+\frac{\partial {\overset{˘}{S}}_{\overset{˘}{X}\overset{˘}{Y}}}{\partial \overset{˘}{Y}}+\left(1-C_{0}\right){g\rho }_{f\zeta_{t}}\left(\overset{˘}{T}-T_{0}\right)$$

(5) $$-g\left(\frac{\rho_{f}-\rho_{p}}{\rho_{f}}\right)\zeta_{c}\left(\overset{˘}{C}-C_{0}\right)-\sigma B_{0}^{2}\overset{˘}{U}-\frac{\mu }{k_{1}}\overset{˘}{U}+k\frac{\partial \overset{˘}{W}}{\partial \overset{˘}{Y}}\text{,}$$

(6) $$\rho_{f}\left[\frac{\partial }{\partial \overset{˘}{t}}+\overset{˘}{U}\frac{\partial }{\partial \overset{˘}{X}}+\overset{˘}{V}\frac{\partial }{\partial \overset{˘}{Y}}\right]\overset{˘}{V}=\frac{\partial }{\partial \overset{˘}{Y}}\left[-\overset{˘}{P}+{\overset{˘}{S}}_{\overset{˘}{Y}\overset{˘}{Y}}\right]+\frac{\partial {\overset{˘}{S}}_{\overset{˘}{Y}\overset{˘}{X}}}{\partial \overset{˘}{X}}-\frac{\mu }{k_{1}}\overset{˘}{V}+k\frac{\partial \overset{˘}{W}}{\partial \overset{˘}{X}}\text{,}$$

$$\left[\frac{\partial }{\partial \overset{˘}{t}}+\overset{˘}{U}\frac{\partial }{\partial \overset{˘}{X}}+\overset{˘}{V}\frac{\partial }{\partial \overset{˘}{Y}}\right]\overset{˘}{T}=\alpha \nabla^{2}\overset{˘}{T}+\frac{1}{\rho_{f}\rho_{p}}\left[{\overset{˘}{S}}_{\overset{˘}{X}\overset{˘}{X}}\frac{\partial \overset{˘}{U}}{\partial \overset{˘}{X}}+{\overset{˘}{S}}_{\overset{˘}{X}\overset{˘}{Y}}\left(\frac{\partial \overset{˘}{U}}{\partial \overset{˘}{Y}}+\frac{\partial \overset{˘}{V}}{\partial \overset{˘}{X}}\right)+{\overset{˘}{S}}_{\overset{˘}{Y}\overset{˘}{Y}}\frac{\partial \overset{˘}{V}}{\partial \overset{˘}{X}}\right]$$

(7) $$-\tau \left[D_{B}\left(\frac{\partial \overset{˘}{C}}{\partial \overset{˘}{X}}\frac{\partial \overset{˘}{T}}{\partial \overset{˘}{X}}+\frac{\partial \overset{˘}{C}}{\partial \overset{˘}{Y}}\frac{\partial \overset{˘}{T}}{\partial \overset{˘}{Y}}\right)+\frac{D_{T}}{T_{m}}\left({\left(\frac{\partial \overset{˘}{T}}{\partial \overset{˘}{X}}\right)}^{2}+{\left(\frac{\partial \overset{˘}{T}}{\partial \overset{˘}{Y}}\right)}^{2}\right)\right]+\frac{\sigma {\overset{˘}{U}}^{2}B_{0}^{2}}{c_{f}\rho_{f}}\text{,}$$

(8) $$\left[\frac{\partial \overset{˘}{C}}{\partial \overset{˘}{t}}+\overset{˘}{U}\frac{\partial \overset{˘}{C}}{\partial \overset{˘}{X}}+\overset{˘}{V}\frac{\partial \overset{˘}{C}}{\partial \overset{˘}{Y}}\right]=D_{B}\nabla^{2}\overset{˘}{C}+\frac{D_{T}}{T_{m}}\nabla^{2}\overset{˘}{T}$$

(9) $$\rho_{f}\overset{˘}{J}\left[\frac{\partial }{\partial \overset{˘}{t}}+\overset{˘}{U}\frac{\partial }{\partial \overset{˘}{X}}+\overset{˘}{V}\frac{\partial }{\partial \overset{˘}{Y}}\right]\overset{˘}{W}=-2k\overset{˘}{W}+\frac{D_{T}}{T_{m}}\nabla^{2}\overset{˘}{W}+k\left(\frac{\partial \overset{˘}{V}}{\partial \overset{˘}{X}}-\frac{\partial \overset{˘}{U}}{\partial \overset{˘}{Y}}\right)$$

Where $\nabla^{2}=\frac{\partial^{2}}{\partial {\overset{˘}{X}}^{2}}+\frac{\partial^{2}}{\partial {\overset{˘}{Y}}^{2}}$, ${\overset{˘}{S}}_{\overset{˘}{X}\overset{˘}{X}},{\overset{˘}{S}}_{\overset{˘}{Y}\overset{˘}{Y}},{\overset{˘}{S}}_{\overset{˘}{X}\overset{˘}{Y}}$ representing extra stress tensor constituents, $\tau =\frac{\left(\rho c\right)p}{\left(\rho c\right)f}$ signifies the ratio of nanoparticles effective heat capacity and fluid heat capacity.

Now relying on wave frame simplifications $\overset{˘}{X}=\overset{˘}{x}+ct,\overset{˘}{U}\left(\overset{˘}{X},\overset{˘}{Y},\overset{˘}{t}\right)=\overset{˘}{u}\left(\overset{˘}{x},\overset{˘}{y}\right),\overset{˘}{T}\left(\overset{˘}{X},\overset{˘}{Y},\overset{˘}{t}\right)=\overset{˘}{T}\left(\overset{˘}{x},\overset{˘}{y}\right)\text{,}$ $\overset{˘}{V}\left(\overset{˘}{X},\overset{˘}{Y},\overset{˘}{t}\right)=\hat{v}\left(\overset{˘}{x},\overset{˘}{y}\right)+c,\hat{p}\left(\overset{˘}{X},\overset{˘}{Y},\overset{˘}{t}\right)=P\left(\overset{˘}{x},\overset{˘}{y}\right),\overset{˘}{W}\left(\overset{˘}{X},\overset{˘}{Y},\overset{˘}{t}\right)=\overset{˘}{w}\left(\overset{˘}{x},\overset{˘}{y}\right)\text{,}$ moreover focusing on the underneath dimensionless parameters

$$x=\frac{\overset{˘}{x}}{\lambda },y=\frac{\overset{˘}{y}}{m_{1}},u=\frac{\overset{˘}{u}}{c},v=\frac{\overset{˘}{v}}{c},t=\frac{c\overset{˘}{t}}{\lambda },h_{1}=\frac{{\overset{˘}{H}}_{1}}{m_{1}},h_{2}=\frac{{\overset{˘}{H}}_{2}}{m_{2}},a=\frac{l_{1}}{m_{1}},b=\frac{l_{2}}{m_{1}}\text{,}$$

$$\delta =\frac{m_{1}}{\lambda \;},P=\frac{m_{1}^{2}\overset{˘}{P}}{c\lambda \mu },M=\sqrt{\frac{\sigma }{\mu }}B_{0}m_{1},\overset{˘}{\lambda_{2}}=\frac{{c\lambda }_{2}}{m_{1}},\mathrm{Pr}=\frac{\nu }{\alpha },Re=\frac{cm_{1}}{\nu },S=\frac{\overset{˘}{S}m_{1}}{\mu c}\text{,}$$

(10) $$G_{m}=\frac{\left(\rho_{c}-\rho_{f}\right)g\zeta_{c}\left(C_{1}-C_{0}\right)m_{1}^{2}\;}{\mu c},N_{t}=\frac{\tau D_{T}\left(T_{1}-T_{0}\right)}{T_{0}\nu },N_{b}=\frac{\tau D_{B}\left(C_{1}-T_{0}\right)}{T_{0}\nu }$$

$$Gr=\frac{\left(T_{1}-T_{0}\right)\rho_{f}g\zeta_{t}\left(1-C_{0}\right)m_{1}^{2}\;}{\mu c},E_{C}=\frac{c^{2}}{\left(T_{1}-T_{0}\right)c_{f}},\eta =\frac{m_{1}^{2}}{k_{1}}=D_{a},Br=E_{c}\;\mathrm{Pr}\text{,}$$

$$\theta =\frac{{\overset{˘}{T}}_{1}-T_{0}}{T_{1}-T_{0}},\Omega =\frac{{\overset{˘}{C}}_{1}-C_{0}}{C_{1}-C_{0}},w=\frac{\overset{˘}{w}m_{1}}{c},J=\frac{\overset{˘}{J}}{m_{1}^{2}}\text{.}$$

Lubrication theory is implemented [[41], [42], [43], [44]] where the aspect ratio (length to width) of the flow region is insignificant. In the context of flows on microchannel, lubrication theory is implemented when the amplitude of the wave is negligible in comparison to the wavelength. These approximations used to simplify the equations of the fluid transport. The lubrication technique, or lubrication theory, is extremely handful in tackling biological fluid problems due to its capacity to simplify the complex system of biological flows where one dimension is significantly smaller than the other. Using wave transformations, non-dimensional parameters and lubrications approximation theory equations of motions for micropolar nanofluid take the underneath form as:

(11) $$\frac{1}{1+\lambda_{1}}\frac{\partial^{2}u}{\partial y^{2}}+Gr\theta +Gm\Omega -\left(M^{2}+\frac{1}{D_{a}}\right)\left(u+1\right)+\frac{k}{\mu }\frac{\partial w}{\partial y}=\frac{\partial p}{\partial x}\text{,}$$

(12) $$-\frac{\partial p}{\partial y}=0\text{,}$$

(13) $$\frac{\partial^{2}\theta }{\partial y^{2}}+Br\left(\frac{1}{1+\lambda_{1}}{\left(\frac{\partial u}{\partial y}\right)}^{2}+M^{2}{\left(\frac{\partial u}{\partial y}+1\right)}^{2}\right)+\mathrm{Pr}\;N_{b}\frac{\partial \Omega }{\partial y}\frac{\partial \theta }{\partial y}+N_{t}\;\mathrm{Pr}\;{\left(\frac{\partial \theta }{\partial y}\right)}^{2}=0\text{,}$$

(14) $$\frac{\partial^{2}\Omega }{\partial y^{2}}+\frac{N_{t}}{N_{b}}\frac{\partial^{2}\theta }{\partial y^{2}}=0\text{,}$$

(15) $$\frac{\gamma }{kd_{1}^{2}}\frac{\partial^{2}w}{\partial y^{2}}-\frac{\partial u}{\partial y}-2w=0\text{,}$$

Respective boundary conditions of the physical flow rheology may be apprised as

(16) $$u_{o}=-1-2\pi \alpha \epsilon \delta \;\cos\;x,\theta =0,\Omega =0,w=0,at\;y=h_{1}=1+a\;\cos\;2\;\pi x$$

(17) $$u_{o}=-1-2\pi \alpha \epsilon \delta \;\cos\;x,\theta =1,\Omega =1,w=0,at\;y=h_{2}=-d-b\;\cos\left(2\pi x+\phi \right)$$

The shear stress at the wall of the microchannel, heat flux, and nanoparticle volume flux may be defined as [35]:

(19) $$C_{f}=\frac{1}{1+\lambda_{1}}{\left[\frac{\partial u}{\partial y}\right]}_{h_{1},h_{2}},Nu={-\left[\frac{\partial \theta }{\partial y}\right]}_{h_{1},h_{2}}Sh=-{\left[\frac{\partial \Omega }{\partial y}\right]}_{h_{1},h_{2}}\text{.}$$

### 2.1 The solution methodology

Now using $u=\frac{\partial \psi }{\partial y}$, equations (9), (10), (11), (12), (13), (14), (15), (16) may be exhibited in term of stream function. Further in order to interrogate the solution of the physical problem let us employ the renowned bvp4c in MATLAB software. The bvp4c is a numerical method vibrantly used in tackling boundary value problems (BVPs). It is based on finite difference method which capitalizes a fourth-order precise scheme to discretize the differential equation with respect to boundary conditions. This method is a robust and extremely reliable method for solving a wide range of BVPs, and is abundantly used in physics, engineering, and problem relating to applied mathematics. In this scheme all the differential equations are transformed into first order equations using simplifications. In order employ the mythology focusing on the transformation as: $\psi =\xi_{1},\psi^{\prime }=\xi_{2},\psi^{\prime \prime }=\xi_{3},\psi^{\prime \prime \prime }=\xi_{4},\theta =\xi_{5},\theta^{\prime }=\xi_{6},\Omega =\xi_{7},\Omega^{\prime }=\xi_{8},w=\xi ,w^{\prime }=\xi_{10}$. Eqs. (9), (10), (11), (12), (13), (14), (15), (16) take the shape as

(20) $$\xi_{4}^{\prime }=\left(1+\lambda_{1}\right)\left[-Gr\xi_{6}-Gm\xi_{8}+\left(M^{2}+\frac{1}{D_{a}}\right)\xi_{3}-\frac{k}{\mu }\xi_{10}^{\prime }\right]\text{,}$$

(21) $$\xi_{6}^{\prime }=-Br\left(\frac{1}{1+\lambda_{1}}{\left(\xi_{3}\right)}^{2}+M^{2}{\left(\xi_{2}+1\right)}^{2}\right)-\mathrm{Pr}\;N_{b}\xi_{8}\xi_{6}-N_{t}\;\mathrm{Pr}\;{\left(\xi_{6}\right)}^{2}\text{,}$$

(22) $$\xi_{8}^{\prime }=-N_{t}N_{b}^{-1}\xi_{6}^{\prime }\text{,}$$

(23) $$\xi_{10}^{\prime }=\frac{kd_{1}}{\gamma_{1}}\left(2\xi_{9}+\xi 3\right)\text{,}$$

(24) $$\xi -\frac{q}{2},\xi_{2}-u_{o},\xi_{5}-0,\xi_{7}-0,\xi_{9}-0,\text{at}\;y=h_{2}$$

(25) $$\xi -\frac{q}{2},\xi_{2}-u_{o},\xi_{5}-1,\xi_{7}-1,\xi_{9},\text{at}\;y=h_{1}$$

## 3 Result and discussion

In this investigation flow dynamic for the micropolar nanofluid in channel carpeted with cilia is elaborated. The flow scheme evolved by maintaining a small difference of temperature and concentration between the walls of the microchannel. The novel mathematical methodology is proposed, and pertinent equation of fluid transport are simplified by employing the lubrication approximation theory. Then equations of motion of the flow in microchannel are tackled with bvp4c scheme. In this juncture of interrogation, we have examined the fluid rheology with the variations different transport parameters like Hartmann number M, Grashof number *Gr*, nanoparticle Grashof number Gm, Darcy number $D_{a}$, Prandtl number Pr, relaxation to retardation parameter $\lambda_{1}$, and viscosity parameter k. In order to carry out quantitative investigation, mathematical approximation is provided for different physical quantities pertaining to physical problems for the transport of rheological fluids under ciliary propulsion. Underneath a range of physical parameters have been utilized [2,14,34].

$$\alpha =0.2,M=\left[0,3\right],\epsilon =0.1,\delta =0.1,a=0.3,b=0.5,\phi =\frac{\pi }{3},\mathrm{Pr}=\left[0.015,21\right]\text{,}$$

$$\lambda_{1}=\left[0.2,0.8\right],D_{a}=\left[0.2,0.8\right],Br=\left[1,4\right],G_{r}=\left[0.5,2\right],G_{m}=\left[0.5,2\right],k=\left[1,4\right]\text{,}$$

$$N_{t}=\left[0.2,0.8\right],\mu =0.5,\gamma_{1}=0.5,d_{1}=0.3$$

### 3.1 Velocity profile

**Fig. 2:**
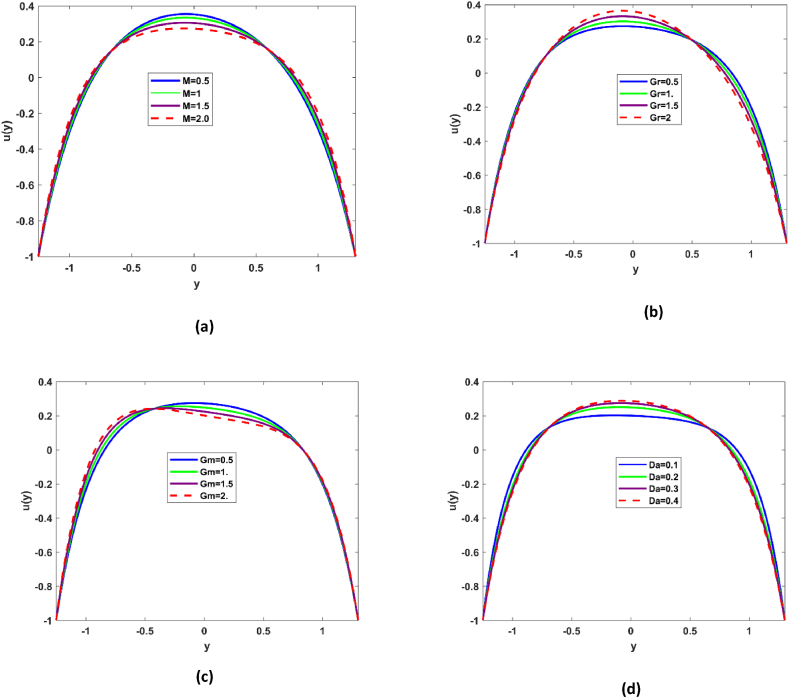
Flow demonstration as variations of Hartmann number M, Grashof number Gr, Nanoparticle Grashof number Gm.

Fig. 1 demonstrate the impact Hartmann No. M, Grashof number Gr, nanoparticle Grashof number Gm and Darcy number on the velocity profile. It has been reported that nanofluid flow transport is affected in the bulk flow of the microchannel due to exclusive dominance of the magnetic properties. Magnetic number depends upon the Lorentz force; rise in the magnetic number refers to surge in the Lorentz force which practically acts as frictional force, that is why decline in the velocity is revealed in Fig. 2(a). Surge in the fluid transport is seen for rising Gr, Grashof number envisages ratio of buoyancy to viscous force. From Fig. 2(b) it may be deduced that in the in bulk of flow regime buoyancy forces are dominant which ultimately give impetus to flow. Decline in the fluid transport phenomena is observed with Nanoparticle Grashof number Gm (see Fig. 2(c)). Darcy number expresses the ratio of permeability of the medium to its cross-sectional area, it is evident that there is decline in the flow along the wall of the channel as permeability phenomena is enhanced, while in the center of the microchannel there is slight inclination in the fluid velocity see Fig. 2(d). This indicates that there is dominance of suction phenomenon at the boundaries.

### 3.2 Temperature profile

**Fig. 3:**
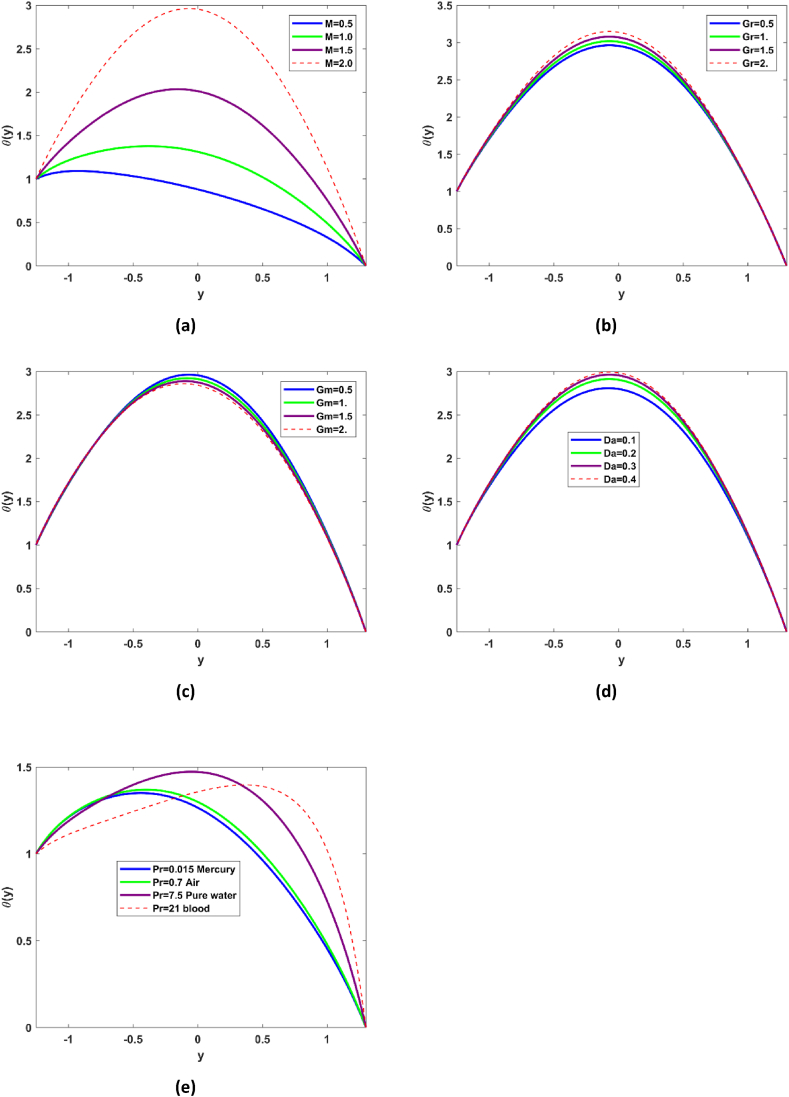
Influence of ,Gr, Gm, $D_{a}$, and Pr on the temperature profile.

Temperature profile variations with Hartmann number M, Grashof number Gr, nanoparticle Grashof number Gm, Darcy number $D_{a}$, and Prandtl number Pr have been elaborated in Fig. No. 3. A substantial rise in the temperature of nanofluid profile is evident from Fig. 3(a) with Hartmann number, as narrated magnetic number relies on the Lorentz force, being resistive force, this gives comprehensive rise to temperature profile. Fig. 3 (b & c) exhibit the appreciable rise in the temperature Gr and quit opposite behavior with Gm. Rise in the temperature profile with Gr indicates that natural convection prevails which increases the temperature of the nanofluid particles. Temperature of the nanofluid is raised with increase in the $D_{a}$ (see Fig. No. 3(d)), which suggest that permeability phenomena provide conducive atmosphere to temperature. In depth analysis of the Prandtl number Pr for the case of mercury, air, pure water, and blood on the temperature profile is examined in the Fig. No. 3(e). Prandtl number is actually the ratio of momentum diffusivity to thermal diffusivity. Substantial rise in the temperature of nanoparticles is revealed for Pr, indicating that thermal diffusivity phenomenon is dominant, it is worth mention here that temperature profile is maximum for the pure water and for blood.

### 3.3 Nanoparticle concentration

**Fig. 4:**
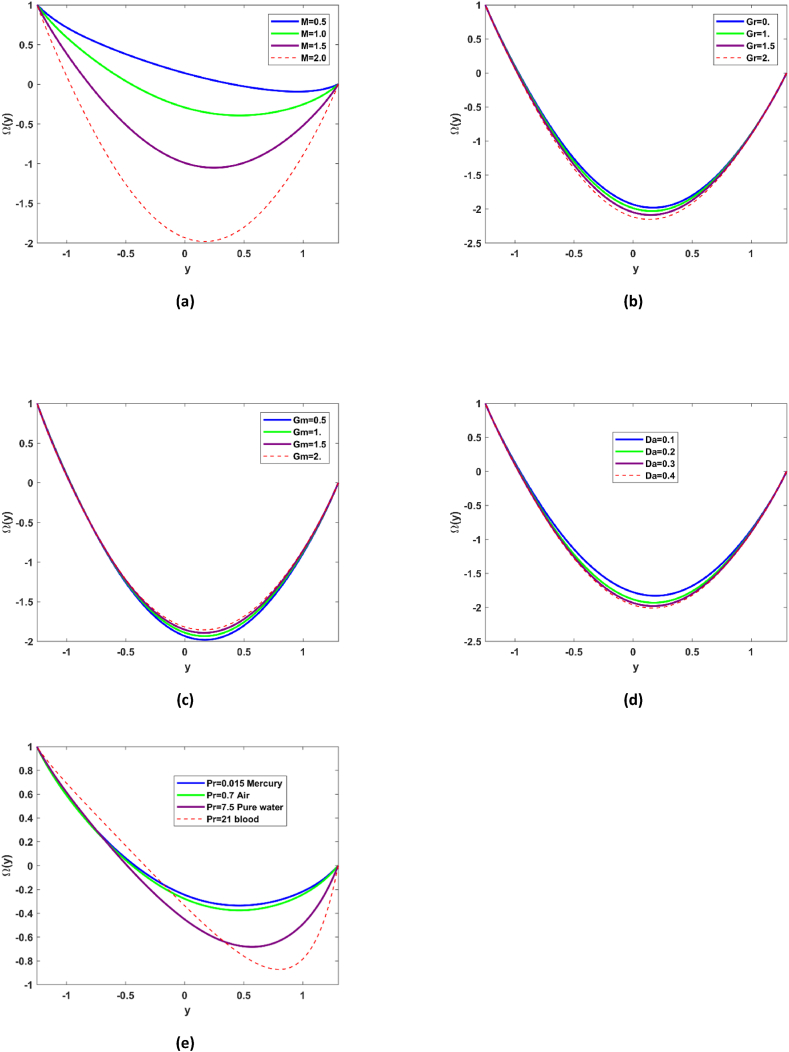
Variations of nanoparticle concentration with different values of ,Gr, Gm, $D_{a}$, and Pr**.**

Thorough investigation of nanoparticle concentration with different values of Hartmann number M, Grashof number *Gr*, nanoparticle Grashof number Gm, Darcy number *Da*, Prandtl number Pr have been elaborated in Fig. 4. A rapid deterioration in the nanoparticle concentration profile of the micropolar nanofluid has been observed with an increasing magnetic field strength (see Fig. 4(a)). Nanoparticle concentration is declined with Gr and reverse trend is evident from Fig. 4(b & c) for Gm and it may be deduced that with enhancement in the buoyancy phenomena diffusion process is slow down with Gr and speed up with Gm. Convection phenomenon is enhanced with Gr which lead to more effective mixing of nanoparticles, potentially resulting in a more consistent nanoparticle concentration throughout the fluid. Fig. 4 (d) reveals that permeability parameter $D_{a}$ affects the diffusion process of nanoparticles concentration. Also, it is worth mentioning here that mercury and air particles have a high rate of diffusion of nanoparticles concentration in comparison to pure water and blood nanoparticles (see Fig. 4 (d)). It is quite evident from Fig. 4(e) as the value of Prandtl number is varied from 0.015 for Mercury to 21 for the blood, the concentration of the nanoparticles significantly declined. It may be concluded that blood particles have the least diffusion phenomena and mercury possesses maximum.

### 3.4 Microrotation profile

**Fig. 5:**
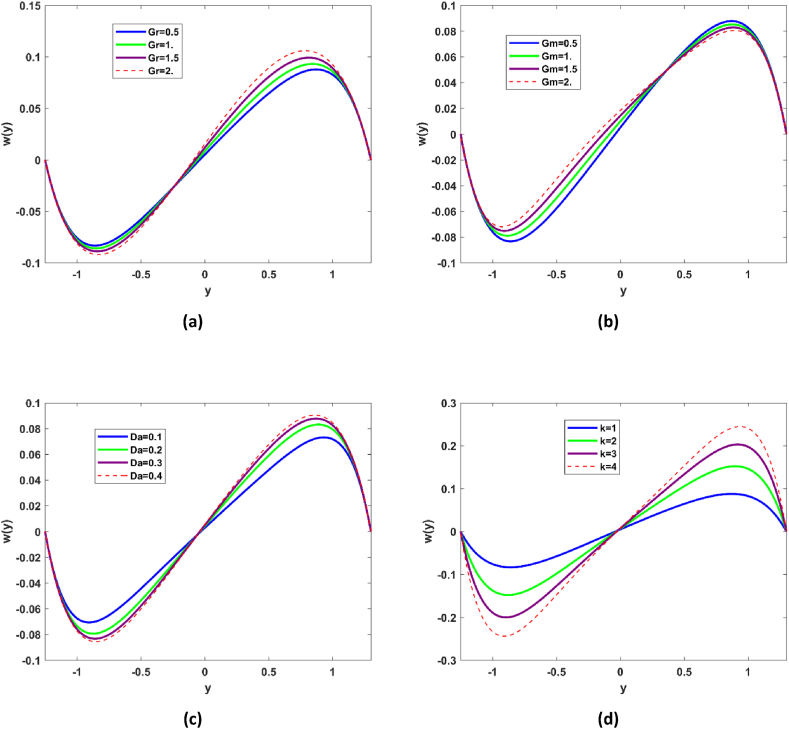
Variations of microrotation of nanofluid with various values of Grashof number Gr, nanoparticle Grashof number Gm, Darcy number $D_{a}$ , viscosity parameter k.

Fig. 5(a–d) demonstrate variations of microrotation profile for different values of Grashof number *Gr*, nanoparticle Grashof number *Gm*, Darcy number *Da*, and viscosity parameter k. As it is discussed earlier that enhancement in Gr gives impetus to convection in Fig. 5(a) it is observed that microrotation of the nanoparticles is also enhanced by Grashof number. It is reported that microrotation of the nanoparticles decreases (see Fig. 5(b)) in flow regime y∈[−1,−0.5] for enhancing the buoyancy forces and surges in y∈[−0.1,1]. Spinning motion of the nanofluid is predominantly enhanced with nanoparticles Grashof number in y∈[−1,0.5] and is decreased in region in region y∈[0.5,1]. Fig. 5(c) illustrate the nanofluid spinning motion for different values of Darcy number and it has been reported there is decline in the microrotation of the nanofluid in region y∈[−1,0] and appreciable rise in the microrotation phenomena in region [0,1] for enhancing the permeability of the ciliated microchannel. There is significant decline in spinning motion of nanofluid with enhancement in the viscosity parameter k, regarding the region y∈[−1,0] and quit opposite trend is evident in region y∈[0,1] refer to Fig. 5(d).

### 3.5 Nusselt number, shear stress variations, pressure gradient

**Fig. 6:**
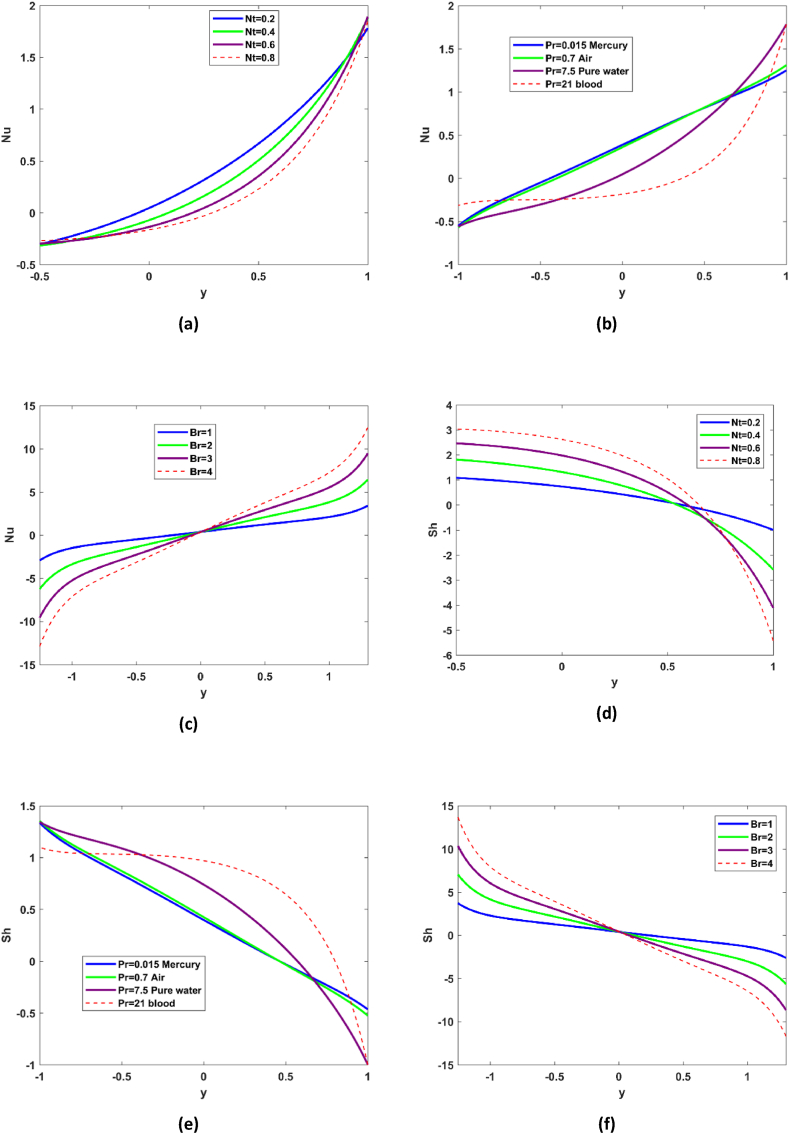
Nusselt number, shear stress variations with $N_{t},\mathrm{Pr},Br$.

**Fig. 7:**
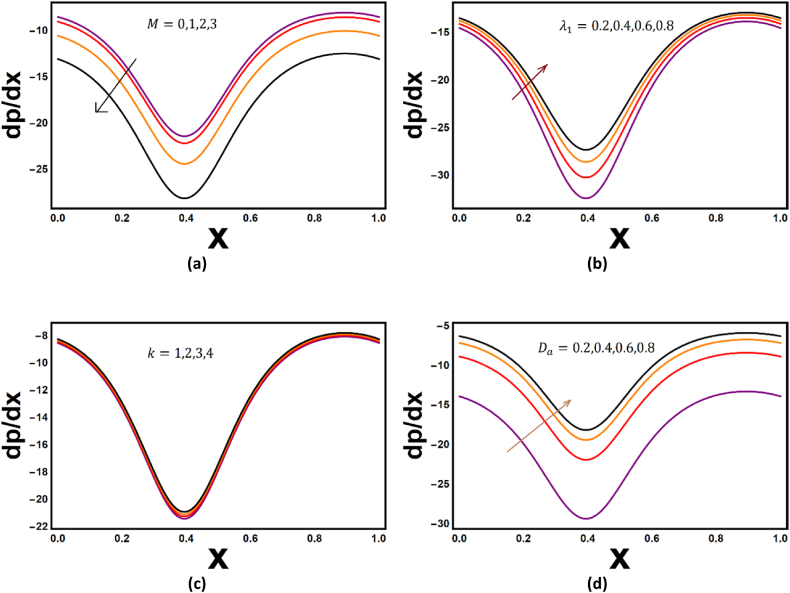
Variations pressure gradient for different values of $M,\lambda_{1},k,D_{a}$.

Effect of different flow indicators have been exhibited for Nusselt number Nu and shear stresses Sh in Fig. 6(a–f). Scientifically speaking the Nusselt number expresses the ratio of heat convection to conduction. An appreciable decline in the Nusselt number is recorded for the thermophoresis parameter $N_{t}$ (see Fig. 6(a)), which suggest that conduction process in enhanced with $N_{t}$. Thermophoresis displaces fluid molecules from hot zone and high energy levels to toward the cold region. Also, there is a decline in the Nusselt number is evident from Fig. 6(b) when the value of Pandtl number is scrutinized for different fluid. From Fig. 6(c) it may be conclude that in region y∈[−1,0] the conduction phenomenon is dominant and in region y∈[0,1] the convection phenomenon is dominant for rising value of Brinkmann number. Fig. 6(d–e) reveals the effect of thermophoresis and Prandtl number on the shear stress, decline in the shear stress is recorded for enhancing $N_{t}$ and rise in seen for Pr. Fig. 6(f) demonstrates mix behavior for shear stress, and it may be concluded that viscous heating is dominant in yϵ[−1,0] and in [0,1] conduction of heat is playing predominate part. Fig. 7(a–c) refers to variations of Hartmann number, relaxation to retardation parameter, permeability parameter and Darcy number. It has been reported that pressure gradient profile decreases to appreciable extent with M and trivially with k (see Fig. 7(a & c)). Pressure gradient is built up for enhancement in relaxation to retardation parameter and Darcy number (see Fig. 7 (b & d)).

### 3.6 Error analysis of BVP4C solution with Artificial Neural Network

**Fig. 8:**
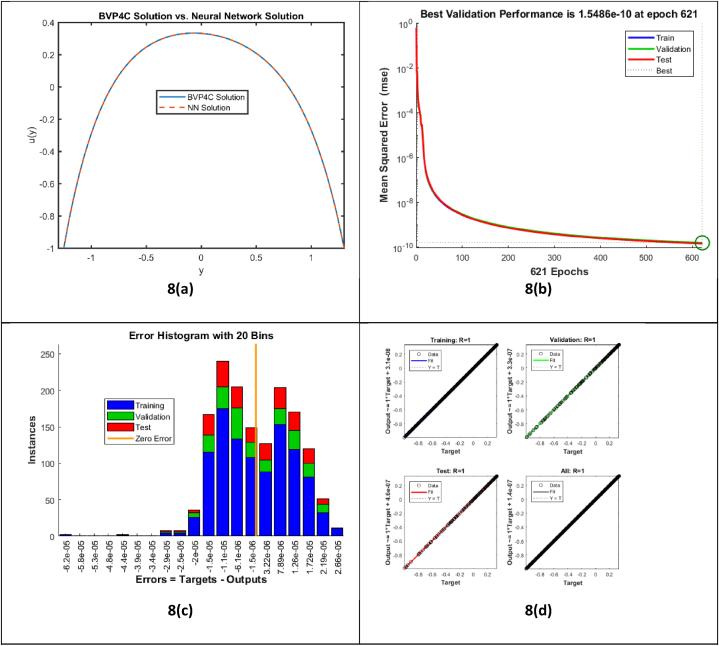
Analysis of BVP4C solution with Artificial Neural Network.

Fig. 8 demonstrates analysis of BVP4C solution with novel Artificial neural network (ANN). It is quite evident from Fig. 8(a) that both solutions are in agreement with each other which validate the proposed solution methodology. Fig. 8(b) exhibits the training process and evaluating the neural network's performance. The best validation mean square error $1.5486\times e^{-10}$ indicates that the neural network has achieved excellent performance, with predictions are in alignment actual target values. Fig. 8(c) infers error histogram provided an in-depth analysis of the neural network's performance by demonstrating the distribution of prediction errors across the training, validation, and test datasets. Showing that the neural network has a good fit to the data, with mostly errors nearly zero and relatively few large errors. Fig. 8(d) shows that the neural network attained perfect performance in all datasets. The agreement of the data points with the ideal line (Y=T) and the correlation coefficient R=1 in each plot reflecting network predictions are highly precise and dependable.

## 4 Conclusion

The flow rheology for the micropolar nanofluid in channel carpeted with cilia is elaborated. A novel mathematical model is presented for heat and mass transfer of MHD micropolar nanofluid transport in an asymmetric channel. The flow inside the microchannel is modelled, and pertinent equation of fluid transport are simplified by employing the lubrication approximation theory. Then equations of motion of the flow in microchannel are tackled with bvp4c scheme. The key findings of this investigation may be summarized as follows.

• The flow transport inside the ciliated microchannel is reinforced with Grashof number and Darcy number whereas quite different scenario is recorded with Magnetic and nanoparticle Grashof numbers.
• Temperature of nanofluid is raised with enhancement in magnetic number, Grashof number, Darcy number, and Prandtl number and goes down with nanoparticle Grashof numbers.
• The diffusion phenomenon of the nanoparticle concentration is decreased with magnetic number, Grashof number, Darcy number, and Prandtl number and escalated with nanoparticle Grashof numbers.
• Spinning motion of the nanofluid is enhanced with Grashof number and slow down with nanoparticle Grashof number and different behavior is recorded for Darcy and viscosity parameters in different flow regime.
• Pressure gradient profile decreases with magnetic number and viscosity parameter whereas, pressure gradient is raised with relaxation to retardation parameter and Darcy number.

The reported investigation provides the knowledge the fluid movement within the efferent ducts of human male reproductive tract. Also, these results will be handful for cilia-based actuators and may prove their significance in micro-pumps employed in various drug-delivery systems. For future studies this work may be extended with power law fluid model, considering shear thinning and thickening behavior of rheological fluid. In future other numerical schemes may be employed like stochastic computing infrastructures etc.

## Data availability statement

The data will be available on suitable request.

## CRediT authorship contribution statement

**Ali Imran:** Writing – original draft, Software, Methodology, Conceptualization. **Hanadi Alzubadi:** Writing – review & editing, Validation, Investigation. **Mohamed R. Ali:** Validation, Supervision, Funding acquisition.

## Declaration of competing interest

The authors unanimously declare that they don't have any conflict of interest.
